# A new unit distribution: properties, estimation, and regression analysis

**DOI:** 10.1038/s41598-024-57390-7

**Published:** 2024-03-27

**Authors:** Kadir Karakaya, C. S. Rajitha, Şule Sağlam, Yusra A. Tashkandy, M. E. Bakr, Abdisalam Hassan Muse, Anoop Kumar, Eslam Hussam, Ahmed M. Gemeay

**Affiliations:** 1 Department of Statistics, Faculty of Sciences, Selcuk University, Konya, Turkey; 2 Department of Mathematics, Amrita School of Physical Sciences, Amrita Vishwa Vidyapeetham, Coimbatore, 641112 India; 3 Department of Statistics and Operations Research, College of Science, King Saud University, P.O. Box 2455, Riyadh, 11451 Saudi Arabia; 4 Faculty of Science and Humanities, School of Postgraduate Studies and Research (SPGSR), Amoud University, Borama, Somalia; 5 Department of Statistics, Faculty of Basic Science, Central University of Haryana, Mahendergarh, 123031 India; 6 Department of Mathematics, Faculty of Science, Helwan University, Cairo, Egypt; 7 Department of Mathematics, Faculty of Science, Tanta University, Tanta, 31527 Egypt

**Keywords:** Stochastic ordering, Monte Carlo simulation, Quantile regression analysis, Beta regression model, Educational attainment dataset, Applied mathematics, Statistics

## Abstract

This research commences a unit statistical model named power new power function distribution, exhibiting a thorough analysis of its complementary properties. We investigate the advantages of the new model, and some fundamental distributional properties are derived. The study aims to improve insight and application by presenting quantitative and qualitative perceptions. To estimate the three unknown parameters of the model, we carefully examine various methods: the maximum likelihood, least squares, weighted least squares, Anderson–Darling, and Cramér-von Mises. Through a Monte Carlo simulation experiment, we quantitatively evaluate the effectiveness of these estimation methods, extending a robust evaluation framework. A unique part of this research lies in developing a novel regressive analysis based on the proposed distribution. The application of this analysis reveals new viewpoints and improves the benefit of the model in practical situations. As the emphasis of the study is primarily on practical applications, the viability of the proposed model is assessed through the analysis of real datasets sourced from diverse fields.

## Introduction

Statistical distributions constitute fundamental mathematical elements in data modeling, inference, and estimating processes, as well as in fields such as public health, actuarial science, biomedical studies, demography, and industrial reliability. Due to the lack of a suitable distribution for the data and the limitations of the existing distribution theory, researchers frequently selected the most appropriate distribution from the available blocks. In many studies, the absence of proper statistical distributions forces researchers in various fields to consistently put effort into developing new distributions to support their judgments. Applied researchers and practitioners often find modeling complex problems to be a perplexing challenge, especially when dealing with diverse lifetime datasets prevalent in physical and natural sciences. In their quest for simplicity and efficiency, exhaustive reviews on this subject can be explored in^1,2^. These references offer comprehensive summaries of statistical distributions derived through various methodologies.

New statistical models built on attractive distributions have long been a favorite in the statistical literature due to the complexity and diversity of modern data. The extended distributions suggested by adding extra parameters provide greater flexibility.

Numerous studies are examined to build probability distributions with substantially more perfect and flexible properties that can model real-life data sets of diverse kinds. The requirement to create new distributions appears from hypothetical concerns, actual applications, or both. There has been a pointed extension in generalizing some well-known distributions and their sensible application to contest more well-known distributions. The exponential distribution is perfect for exposing the life data, like for many types of industrial items. The major highlight of the exponential distribution is that it may be used to model the performance of objects with a fixed failure rate. The primary objective of this paper is to present a new, better model capable of modeling and fitting distinct forms of data. It also aims to exhibit the dominance of the new model in surpassing every opponent. It proposes a new model as a strong and novel contestant for modeling real data sets. Once demonstrating a situation with a known model is difficult, we might use generalization to account for extra data variation. The challenges present at this time are progressing significantly along with our world. As of this, we insist on extra generalizations of probability distributions to capture more complicated data. Also, it might be used to analyze many real-life data sets and fit them quite well; it can also be used in various problems in applied areas such as medicine, engineering, and industrial reliability analysis.

Moreover, numerous families of probability distribution have been suggested by a combining technique tracking the innovative work of Adamidis and Loukas^3^. Composite types have been headed in the situation of reliability study when the lifespan can be declared as the least or extreme of a system of independent and identically distributed (i.i.d.) random variables demonstrating system components failure times. The new combination of distributions can extend well-known classical distributions and provide flexibility in modeling data. Combining some valid lifetime data with power series (PS) distributions has been proposed by quite a few authors. Some of them are exponential-PS, Weibull-PS, generalized exponential PS, extended Weibull PS, Burr XII PS, Lindley PS, generalized inverse Weibull PS, and complementary exponentiated inverted Weibull PS distributions^4–11^.

Also, the power function (PF) distribution is a flexible lifetime distribution that may offer a suitable fit to some sets of failure data. Some generalized distributions from PF are beta PF^12^, Weibull^13^, Kumaraswamy PF^14^, transmuted PF (TPF)^15^, exponentiated Kumaraswamy PF^16^, exponentiated Weibull PF^17^ and odd generalized exponential PF^18^. In addition to the above-mentioned distributions some nonlinear predictive network epidemic models were introduced in the literature^19–23^

The primary objective of this article is to introduce an advanced model designed for the modeling and fitting of data defined on (0,1). We aim to demonstrate the superiority of this new model by surpassing all existing competitors. We advocate for the proposed distribution as a robust and innovative choice for modeling real datasets. In situations where modeling with a known distribution proves challenging, the utilization of generalization becomes crucial to accommodate additional variations in the data.

This paper aims to develop a three-parameter alternative to several lifetime distributions, including the Kumaraswamy^24^, unit-Weibull^25^, unit-Burr XI^26^, unit-Muth^27^, and new power function^28^ distributions. In this context, we propose and develop the statistical properties of the proposed distribution and show that it is a better model for reliability analysis to the data defined on (0,1).

In this paper, a new extended form of the new power function distribution (NPFD) is proposed using the power transformation $X=T^{\frac{1}{\sigma }}$ is applied to the cumulative distribution function (CDF) of NPFD. The proposed distribution is called the power new power function distribution (PNPFD). The PNPFD provides increasing, bathtub, J-shaped, reverse J-shaped, and decreasing shapes. Its density can be left-skewed, unimodal, right-skewed, concave down, or constant. Furthermore, this paper aims to delve into the main statistical properties of the PNPFD distribution. The analysis encompasses the shapes of the density function and hazard rate function, moments, incomplete moments, moment generating function (MGF), order statistics, stochastic ordering, and parameter estimation through the maximum likelihood method. To underscore the practical utility of the model, applications to real datasets are provided, demonstrating the distribution’s applicability and usefulness.

An investigation of the relationship between independent one or more variables and the dependent variable is conducted by a classical regression model. The classical regression models correlate the mean response by giving specific values of the independents. In cases where the dependent variable contains an outlier, the classical regression models can be insufficient. The median can handle these scenarios better than the mean since it is a more robust estimate. For these cases, many quantile regression models were introduced such as the beta regression model by^29^, the Kumaraswamy regression model by^30^, unit Weibull regression model by^25^, unit Burr-XII regression model by^26^, the unit Burr-Hatke regression model by^31^, the unit log-log regression model by^32^, etc. This paper also introduces a new quantile regression model as an alternative to current ones based on the proposed distribution.

In this paper, we propose a new distribution as a novel probability distribution model tailored for data defined on the interval (0,1), This study makes a significant contribution to the field of statistics by thoroughly examining its statistical and reliability features. By discussing moments, stochastic ordering, reliability function, hazard rate function, order statistics, and quantile function, we comprehensively understand the PNPFD’s properties. Furthermore, we establish a framework for comparing the efficacy of the PNPFD against selected distributions like the Kumaraswamy and beta distributions. This comparative analysis sets the stage for evaluating the PNPFD’s performance in various statistical applications. Through rigorous parameter estimation techniques and Monte Carlo simulations, we demonstrate the precision and reliability of the PNPFD in handling real-world data. Additionally, introducing a novel regression analysis technique based on the PNPFD expands the scope of statistical modeling, particularly in scenarios where the dependent variable is proportional. Overall, this study presents a new distribution model and highlights its potential to enhance statistical analyses across diverse domains.

The rest of the paper is organized as follows: Section 2(Model formulation) introduces the nature of the probability density function (PDF) and hazard rate function (HRF) of the PNPFD. Its associated statistical properties, such as the moment generating function (mgf), moments, MRL, order statistics, stochastic ordering, and quantile function are investigated in Sect. 3(Statistical properties). The estimation of the parameters is discussed in Sect. 4(Estimation methods). The significant sample behavior of the PNPFD, with the help of certain simulated data sets, is detailed in Sect. 5(Numerical simulation). In Sect. 6(Regression analysis), a novel quantile regression is presented based on PNPFD. In Sect. 7(Real data analysis), real data sets are analyzed using the proposed distribution. Finally, the study is concluded in 8.

## Model formulation

In (2021), Iqbal et al.^28^ derived a new statistical model called new power function distribution (NPFD) with CDF defined as follows

1 $$\begin{aligned} G(t)=1-\left( \frac{1-t}{\delta t+1}\right) ^{\eta },\quad ~0<t<1,~\eta >0,~-1<\delta <\infty . \end{aligned}$$

its PDF is defined as follows

2 $$\begin{aligned} g(t)=(\delta +1) \eta (1-t)^{\eta -1} (\delta t+1)^{-\eta -1}. \end{aligned}$$

The power transformation $X=T^{\frac{1}{\sigma }}$ is applied to the CDF (1) to have power new power function distribution (PNPFD) with CDF defined as follows

3 $$\begin{aligned} F(x)=1-\left( \frac{1-x^{\sigma }}{\delta x^{\sigma }+1}\right) ^{\eta },~0<x<1,~\eta ,~\sigma >0,~-1<\delta <\infty . \end{aligned}$$

we have PNPFD PDF defined as follows

4 $$\begin{aligned} f(x)=\frac{(\delta +1) \eta \sigma x^{\sigma -1} \left( \frac{1-x^{\sigma }}{\delta x^{\sigma }+1}\right) ^{\eta }}{\left( 1-x^{\sigma }\right) \left( \delta x^{\sigma }+1\right) }. \end{aligned}$$

**Figure 1:**
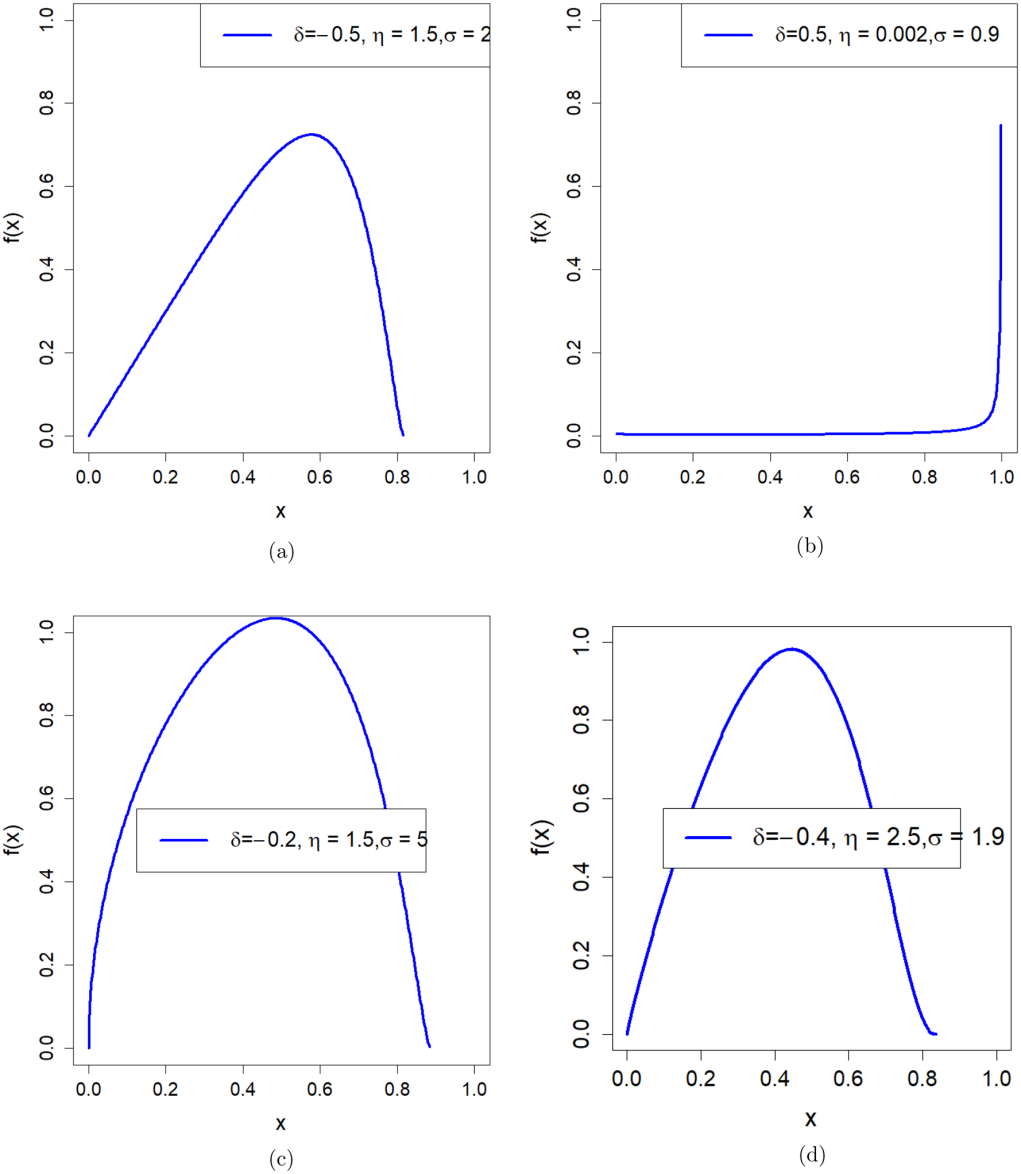
Plot for PDF of the PNPFD for different parameters values.

Figure 1 shows the graphical representation of the PDF of the PNPFD for different combinations of parameter values of $\delta$, $\eta$, and $\sigma$. Figure 1a–d show that they can be unimodal with monotonically increasing and then decreasing for some parameter combinations. Figure 1b shows a constant trend initially, increasing rapidly as *x* increases (J-shaped), and Fig. 1a shows that it can be skewed to the left. Figure 1c,d show that the PDF of PNPFD can be symmetric.

## Statistical properties

### Mixture representation

The expansion of the PDF of the PNPFD proves valuable in deriving its properties. To facilitate this, we employ the following two lemmas:

#### Lemma 1

*If* $\lambda$ *is a positive real non-integer and* $\mid y \mid \le 1$, *from Gradshteyn et al.*^33^ *Equation (1.110) we get binomial series expansion as;*

$$\begin{aligned} (1-y)^{\lambda -1} = \sum _{i=0}^{\infty }(-1)^i {\left( {\begin{array}{c}\lambda -1\\ i\end{array}}\right) }y^i. \end{aligned}$$

#### Lemma 2

*If a is a positive real non-integer and* $\mid y^b \mid > 1$

$$\begin{aligned} (1+y^b)^{-a} = \sum _{k=0}^{\infty } {\left( {\begin{array}{c}a+k-1\\ k\end{array}}\right) }y^{-b(k+a)}. \end{aligned}$$

*and If a is a positive real non-integer and* $\mid y^b \mid < 1$

$$\begin{aligned} (1+y^b)^{-a} = \sum _{k=0}^{\infty } {\left( {\begin{array}{c}a+k-1\\ k\end{array}}\right) }y^{bk}. \end{aligned}$$

Using Lemmas 1 and  2, the expansion of PDF of the PNPFD can be derived as follows.

Case I: $0<\delta x^{\sigma }<1$, we have

5 $$\begin{aligned} f(x)=(\delta +1)\delta ^k \eta \sigma \sum _{j=0}^{\infty } \sum _{k=0}^{\infty } (-1)^{j+k}{\left( {\begin{array}{c}\eta -1\\ j\end{array}}\right) } {\left( {\begin{array}{c}\eta +k\\ k\end{array}}\right) }x^{\sigma (j+k+1) -1.} \end{aligned}$$

Case II: $\delta x^{\sigma }>1$, we have

6 $$\begin{aligned} f(x)=(\delta +1)\delta ^{-(k+\eta +1)}\eta \sigma \sum _{j=0}^{\infty } \sum _{k=0}^{\infty } (-1)^{j+k}{\left( {\begin{array}{c}\eta -1\\ j\end{array}}\right) } {\left( {\begin{array}{c}\eta +k\\ k\end{array}}\right) }x^{\sigma (j-k-\eta )-1}. \end{aligned}$$

### Reliability characteristics of the PNPFD

The reliability function (rf) of the PNPFD is given by

7 $$\begin{aligned} R(x)=\left[ \frac{(1-x^{\sigma })}{(\delta x^{\sigma }+1)}\right] ^\eta . \end{aligned}$$

The HRF of the PNPFD is given by

8 $$\begin{aligned} H(x)=\frac{(\delta +1) \eta \sigma x^{\sigma -1} }{(1-x^{\sigma })(\delta x^{\sigma }+1)}. \end{aligned}$$

**Figure 2:**
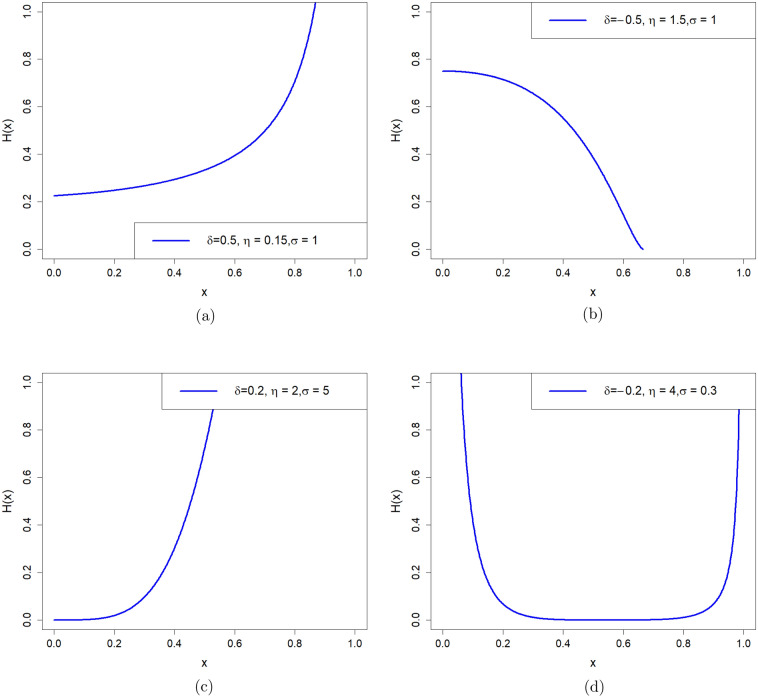
Plot for HRF of the PNPFD for different parameters values.

Figure 2 gives examples of the shapes of the hazard function of our proposed model for different values of $\delta$, $\eta$, and $\sigma$. Figure 2a,c show that the hazard rate function of PNPFD can be increased. Figure 2b shows that the hazard rate function can be decreased, and Fig. 2d shows that the hazard rate function of PNPFD is bathtub-shaped, depending on the values of its parameters.

The reverse hazard rate function (rhrf) of the PNPFD is given by

9 $$\begin{aligned} W(x)=\frac{\eta \sigma (\delta +1) x^{\sigma -1}(1-x^{\sigma })^{\eta -1} }{(\delta x^{\sigma }+1)\left[ (\delta x^{\sigma }+1)^\eta -(1-x^{\sigma })^\eta \right] }. \end{aligned}$$

### Moments

*The* $r^{th}$ *moment* $E(X^r)$ *of PNPFD is given by*

Case I: $$0<\delta x^{\sigma }<1$$

10 $$\begin{aligned} E(X^r) =\frac{(\delta +1)\delta ^k \eta \sigma \sum _{j=0}^{\infty } \sum _{k=0}^{\infty } (-1)^{j+k}{\left( {\begin{array}{c}\eta -1\\ j\end{array}}\right) } {\left( {\begin{array}{c}\eta +k\\ k\end{array}}\right) }}{\sigma (j+k+1)+r}. \end{aligned}$$

Case II: $$\delta x^{\sigma }>1$$,

11 $$\begin{aligned} E(X^r) =\frac{(\delta +1)\delta ^{-(k+\eta +1)}\eta \sigma \sum _{j=0}^{\infty } \sum _{k=0}^{\infty } (-1)^{j+k}{\left( {\begin{array}{c}\eta -1\\ j\end{array}}\right) } {\left( {\begin{array}{c}\eta +k\\ k\end{array}}\right) }}{\sigma (j-k-\eta )+r}. \end{aligned}$$

The first four moments of the PNPFD are obtained by substituting $r=1,2,3,4$ in Eqs. (10) and (11)

### Moment generating function

The MGF of a PNPFD random is given by, Case I: $0<\delta x^{\sigma }<1$

$$\begin{aligned} M_X(t) = \sum _{r=0}^{\infty }\frac{t^r}{r!}\mu _r' = \frac{(\delta +1)\delta ^k \eta \sigma }{\sigma (j+k+1)+r}\sum _{r=0}^{\infty }\frac{t^r}{r!} \sum _{j=0}^{\infty } \sum _{k=0}^{\infty } (-1)^{j+k}{\left( {\begin{array}{c}\eta -1\\ j\end{array}}\right) } {\left( {\begin{array}{c}\eta +k\\ k\end{array}}\right) }. \end{aligned}$$

Case II: $$\delta x^{\sigma }>1$$

$$\begin{aligned} M_X(t) = \sum _{r=0}^{\infty }\frac{t^r}{r!}\mu _r' = \frac{(\delta +1)\delta ^{-(k+\eta +1)}\eta \sigma }{\sigma (j-k-\eta )+r}\sum _{r=0}^{\infty }\frac{t^r}{r!} \sum _{j=0}^{\infty } \sum _{k=0}^{\infty } (-1)^{j+k}{\left( {\begin{array}{c}\eta -1\\ j\end{array}}\right) } {\left( {\begin{array}{c}\eta +k\\ k\end{array}}\right) }. \end{aligned}$$

### Incomplete moment

The incomplete $r^{th}$ moment is defined by

$$\begin{aligned} m_r(x) = \int _{0}^{x} x^r f(x) dx. \end{aligned}$$

For the PNPFD, incomplete $r^{th}$ moment is obtained by;

Case I: $$0<\delta x^{\sigma }<1$$

12 $$\begin{aligned} \begin{aligned} m_r(x) = \frac{(\delta +1)\delta ^k \eta \sigma }{\sigma (j+k+1)+r} \sum _{j=0}^{\infty } \sum _{k=0}^{\infty } (-1)^{j+k}{\left( {\begin{array}{c}\eta -1\\ j\end{array}}\right) } {\left( {\begin{array}{c}\eta +k\\ k\end{array}}\right) }x^{\sigma (j+k+1)+r}. \end{aligned} \end{aligned}$$

Case II: $$\delta x^{\sigma }>1$$

13 $$\begin{aligned} \begin{aligned} m_r(x) = \frac{(\delta +1)\delta ^k \eta \sigma }{\sigma (j-k-\eta )+r} \sum _{j=0}^{\infty } \sum _{k=0}^{\infty } (-1)^{j+k}{\left( {\begin{array}{c}\eta -1\\ j\end{array}}\right) } {\left( {\begin{array}{c}\eta +k\\ k\end{array}}\right) }x^{\sigma (j-k-\eta )+r}. \end{aligned} \end{aligned}$$

### Mean residual life function

The mean residual life (MRL) function is significant in reliability and survival analysis. It describes how long a system will operate, beginning at the time *x*. For PNPFD, the MRL is obtained as,

14 $$\begin{aligned} \phi (x) =\frac{R(x+t)}{R(t)} =\Bigg [\frac{(\delta x^{\sigma }+1)(1-(x+t)^{\sigma })}{(\delta (x+t)^{\sigma } +1)(1-(x)^{\sigma })}\Bigg ]^\eta . \end{aligned}$$

### PDF and CDF of order statistics

The order statistics of a distribution are derived by arranging the sample values in ascending order. The PDF of the $r^{th}$ order statistic is expressed as:

$$\begin{aligned} f_{r:n}(x) = C_{r:n} [F(x)]^{r-1}[1-F(x)]^{n-r}f(x). \end{aligned}$$

where, $$C_{r:n}= \frac{n!}{(r-1)! (n-r)!}$$

Using Eqs. (3) and (4),the PDF of the $r^{th}$ the order statistic of PNPFD is given as

15 $$\begin{aligned} \begin{aligned} f_{r:n}(x) =C_{r:n} (\delta +1) \eta \sigma \Bigg [1-\left( \frac{1-x^{\sigma }}{\delta x^{\sigma }+1}\right) ^{\eta }\Bigg ]^{r-1 }(1-x^{\sigma })^{\eta (n-k+1)-1}(\delta x^{\sigma }+1)^{-\eta (n-k+1)-1} \end{aligned}. \end{aligned}$$

Moreover, the CDF of $r^{th}$ the order statistic is given as

$$\begin{aligned} F_{r:n}(x) = \sum _{m=r}^{n}C_{n:m} [F(x)]^{m}[1-F(x)]^{n-m}. \end{aligned}$$

Using Eq. (3) the CDF of the $r^{th}$ the order statistic of PNPFD is given as

$$\begin{aligned} F_{r:n}(x) = \sum _{m=r}^{n}\left( {\begin{array}{c}n\\ m\end{array}}\right) \Bigg [1-\left( \frac{1-x^{\sigma }}{\delta x^{\sigma }+1}\right) ^{\eta }\Bigg ]^{m}\Bigg [\frac{1-x^{\sigma }}{\delta x^{\sigma }+1}\Bigg ]^{\eta (n-m)} \end{aligned}$$

### Stochastic ordering

For a random variable *X* to be smaller than a random variable *Y*, certain conditions must be satisfied:

(i) Hazard rate order $X \le _{hr} Y$ if $h_X(x) \ge h_Y(x)$
(ii) Stochastic order $X \le _{st} Y$ if $F_X(x) \ge F_Y(x)$
(iii) Mean residual life order $X \le _{mrl} Y$ if $M_X(x) \le M_Y(x)$
(iv) Likelihood ratio order $X \le _{lr} Y$ if $\frac{f_X(x)}{f_Y(x)}$ decreasing in *x*

#### Theorem 1

*Let random variables* $X \sim PNPFD(\sigma _1,\eta _1,{\delta }_{1}) and Y \sim PNPD(\sigma _2,\eta _2,{\delta }_{2})$ *and if* $\sigma _1 \le \sigma _2,\eta _1 \le \eta _2, \delta _1 \le {\delta }_2,$* we have* $X \le _{lr} Y$ *then* $X \le _{hr} Y, X \le _{mr} Y$ *and* $X \le _{st} Y$

#### Proof

To prove $\frac{f_X(x)}{f_Y(x)}$ decreasing in *x* we have to show that the derivative of $\frac{f_X(x)}{f_Y(x)}$ is less than 0.

$$\begin{aligned} \begin{aligned} \frac{f_X(x)}{f_Y(x)} =\frac{\frac{(\delta _1 +1) \eta _1 \sigma _1 x^{\sigma _1 -1} \left( \frac{1-x^{\sigma _1 }}{\delta _1 x^{\sigma _1 }+1}\right) ^{\eta _1 }}{\left( 1-x^{\sigma _1 }\right) \left( \delta _1 x^{\sigma _1 }+1\right) }}{\frac{(\delta _2 +1) \eta _2 \sigma _2 x^{\sigma _2 -1} \left( \frac{1-x^{\sigma _2}}{\delta _2 x^{\sigma _2 }+1}\right) ^{\eta _2}}{\left( 1-x^{\sigma _2}\right) \left( \delta _2 x^{\sigma _2 }+1\right) }}. \end{aligned} \end{aligned}$$

To prove $\frac{f_X(x)}{f_Y(x)}$ is less than 0, we can also show that the derivative of the logarithm of $\frac{f_X(x)}{f_Y(x)}$ is less than 0.

16 $$\begin{aligned} \begin{aligned} \frac{d}{dx} ln\big (\frac{f_X(x)}{f_Y(x)}\big ) =\frac{\sigma _1-\sigma _2}{x}-\sigma _1x^{\sigma _1-1} \Bigg [\frac{\eta _1-1}{1-x^{\sigma _1}}+\delta _1\frac{\eta _1+1}{1+\delta _1x^{\sigma _1}}\bigg ]+\sigma _2x^{\sigma _2-1} \Bigg [\frac{\eta _2-1}{1-x^{\sigma _2}}+\delta _2 \frac{\eta _2+1}{1+\delta _2x^{\sigma _2}}\bigg ]. \end{aligned} \end{aligned}$$

which is less than 0, when $\sigma _1 \le \sigma _2,\eta _1 \le \eta _2, \delta _1 \le {\delta }_2$. Hence, we proved $Y \ge _{lr} X$ so we can say that $Y \ge _{hr} X, Y \ge _{mrl} X$ and $Y \ge _{st} X$ when *Y* and *X* follows the PNPFD. $\square$

### Quantile function

By obtaining the CDF (3) of the PNPFD, the quantile function (QF) of the PNPFD is obtained by calculating the inverse function of the CDF (3) as follows

17 $$\begin{aligned} Q(p)=\left( -\frac{(1-p)^{1/\eta }-1}{\delta (1-p)^{1/\eta }+1}\right) ^{1/\sigma },\quad 0<p<1. \end{aligned}$$

## Estimation methods

In this section, many estimators like maximum likelihood, least squares, weighted least squares, Anderson–Darling, and Cramér-von Mises are examined to estimate the parameters $\sigma , \eta$ and $\delta$ of PNPFD. Let $X_{1},X_{2},\ldots ,X_{n}$ be a random sample from the $PNPFD\left( \sigma ,\eta ,\delta \right)$ distribution and $x_{1},x_{2},\ldots ,x_{n}$ represents the values of the sample. Let $X_{\left( 1\right) },X_{\left( 2\right) },\ldots ,X_{\left( n\right) }$ represent the order statistics for sample $X_{1},X_{2},\ldots ,X_{n}$ with realization $x_{\left( 1\right) },x_{\left( 2\right) },\ldots ,x_{\left( n\right) }$. The likelihood and log-likelihood functions can be given as

$$\begin{aligned} L\left( \Xi \right) =\left( 1+\delta \right) ^{n}\left( \eta \sigma \right) ^{n}\prod \limits _{i=1}^{n}\frac{x_{i}^{-1+\sigma }\left( \frac{ 1-x_{i}^{\sigma }}{1+x_{i}^{\sigma }\delta }\right) ^{\eta }}{\left( 1-x_{i}^{\sigma }\right) \left( 1+x_{i}^{\sigma }\delta \right) }. \end{aligned}$$

and

$$\begin{aligned} \ell \text { }\left( \Xi \right)= & {} n\log \left( 1+\delta \right) +n\log \left( \eta \right) +n\log \left( \sigma \right) +\left( \sigma -1\right) \sum \limits _{i=1}^{n}\log \left( x_{i}\right) \\{} & {} +\eta \text { }\sum \limits _{i=1}^{n}\log \left( \frac{1-x_{i}^{\sigma }}{ 1+x_{i}^{\sigma }\delta }\right) -\sum \limits _{i=1}^{n}\log \left( 1-x_{i}^{\sigma }\right) -\sum \limits _{i=1}^{n}\log \left( 1+x_{i}^{\sigma }\delta \right) . \end{aligned}$$

where $\Xi =\left( \sigma ,\eta ,\delta \right)$. The maximum likelihood estimates(MLE) of $\Xi$, say, ${\widehat{\Xi }}=\left( \widehat{ \sigma },{\widehat{\delta }},{\widehat{\eta }}\right)$ is obtained as follows:

$$\begin{aligned} {\widehat{\Xi }}=\underset{\left( \sigma ,\eta ,\delta \right) \in \left( 0,\infty \right) \times \left( 0,\infty \right) \times \left( -1,\infty \right) }{\arg \max \ell \text { }\left( \Xi \right) }. \end{aligned}$$

Let us deal with the following five functions to obtain the other estimators:

18 $$\begin{aligned} LS\left( \Xi \right)= & {} \sum \limits _{i=1}^{n}\left( \left( 1-\left( \frac{ 1-x_{\left( i\right) }^{\sigma }}{1+x_{\left( i\right) }^{\sigma }\delta } \right) ^{\eta }\right) -\frac{i}{n+1}\right) ^{2} . \end{aligned}$$

19 $$\begin{aligned} WLS\left( \Xi \right)= & {} \sum \limits _{i=1}^{n}\frac{\left( n+2\right) \left( n+1\right) ^{2}}{i\left( n-i+1\right) }\left( \left( 1-\left( \frac{ 1-x_{\left( i\right) }^{\sigma }}{1+x_{\left( i\right) }^{\sigma }\delta } \right) ^{\eta }\right) -\frac{i}{n+1}\right) ^{2} . \end{aligned}$$

20 $$\begin{aligned} AD\left( \Xi \right)= & {} -n-\sum \limits _{i=1}^{n}\frac{2i-1}{n}\log \left\{ \left( 1-\left( \frac{1-x_{\left( i\right) }^{\sigma }}{1+x_{\left( i\right) }^{\sigma }\delta }\right) ^{\eta }\right) \right\} \nonumber \\{} & {} +\log \left\{ \left( \frac{1-x_{\left( n+i-1\right) }^{\sigma }}{ 1+x_{\left( n+i-1\right) }^{\sigma }\delta }\right) ^{\eta }\right\} . \end{aligned}$$

and

21 $$\begin{aligned} CvM\left( \Xi \right) =\frac{1}{12n}+\sum \limits _{i=1}^{n}\left[ \left( 1-\left( \frac{1-x_{\left( i\right) }^{\sigma }}{1+x_{\left( i\right) }^{\sigma }\delta }\right) ^{\eta }\right) -\frac{2i-1}{2n}\right] ^{2}. \end{aligned}$$

The least-square estimates (*LSEs*), weighted least square estimate (*WLSEs*), Anderson–Darling estimate (*ADEs*) and Cramér–von Mises estimate (*CvMEs*) are achieved by minimizing Eqs. (18)–(21), respectively.

## Numerical simulation

In this section, the bias and mean squared errors (MSEs) of MLEs, LSEs, WLSEs, ADEs, and CvMEs for parameters of the PNPFD are obtained via 5000 runs. For generating samples for the PNPFD in the simulation experiment, the quantile function provided in Eq. (17) is used. Furthermore, optimization procedures for obtaining estimations from the generated samples are performed using the BFGS method in the **optim** function in R. Six different scenarios are evaluated for parameter settings. These are $\Xi _{1}=\left( 0.5,1.5,-0.5\right)$, $\Xi _{2}=\left( 2,1.5,-0.5\right) ,$ $\Xi _{3}=\left( 1.5,0.5,2\right)$, $\Xi _{4}=\left( 3,1.5,2\right) ,$ $\Xi _{5}=\left( 0.5,2.5,-0.7\right)$ and $\Xi _{6}=\left( 2.5,0.7,1.5\right)$. The simulation results are given in Tables 1 and 2. Tables 1 and 2 show that the bias and MSEs decrease as the sample size increases for all estimators. According to the bias criterion, the best estimator for the parameters of $\sigma$ and $\eta$ is usually ADEs, while the best estimator for the $\delta$ parameter is MLEs. When scenarios are analyzed in detail, the following interpretations can be made for the MSEs criterion:

- In scenario $\Xi _{1}$, the MLEs for $\sigma$ and ADEs for both $\eta$ and $\delta$ are the best estimators.
- In scenario $\Xi _{2}$, the LSEs for $\sigma$ and CVMEs for both $\eta$ and $\delta$ are the best estimators.
- In scenarios $\Xi _{3}$ and $\Xi _{6}$, the WLSEs are the best estimators for three parameters.
- In scenarios $\Xi _{4}$ and $\Xi _{5}$, the MLEs for $\sigma$ and ADEs for both $\eta$ and $\delta$ are the best estimators.

It is observed that the decreasing trend in bias and MSEs for all estimators is achieved as expected with the increase in sample size.

**Table 1:** The bias of all estimators for PNPFD.

*n*	$\Xi$	$\sigma$					$\eta$					$\delta$				
		*MLEs*	*LSEs*	*WLSEs*	*ADEs*	*CVMEs*	*MLEs*	*LSEs*	*WLSEs*	*ADEs*	*CVMEs*	*MLEs*	*LSEs*	*WLSEs*	*ADEs*	*CVMEs*
50	$\Xi _{1}$	0.0451	0.2395	0.6963	0.0341	0.5279	0.0658	0.5360	0.4422	− 0.0200	0.2547	0.0269	0.3933	0.4262	0.0361	0.3282
75		0.0262	0.1241	0.3452	0.0220	0.3220	0.0426	0.3280	0.3386	− 0.0216	0.1343	0.0166	0.2094	0.2458	0.0218	0.1829
100		0.0247	0.1042	0.2099	0.0198	0.2410	0.0351	0.2432	0.2592	− 0.0133	0.1137	0.0161	0.1584	0.1615	0.0206	0.1448
125		0.0200	0.0806	0.1263	0.0121	0.1690	0.0241	0.1702	0.2020	− 0.0117	0.0887	0.0118	0.1138	0.1093	0.0156	0.1090
150		0.0148	0.0587	0.1109	0.0084	0.1297	0.0184	0.1307	0.1712	− 0.0126	0.0660	0.0076	0.0840	0.0930	0.0108	0.0808
175		0.0102	0.0473	0.0987	0.0049	0.1084	0.0134	0.1091	0.1532	− 0.0139	0.0539	0.0040	0.0678	0.0858	0.0071	0.0667
200		0.0098	0.0416	0.0813	0.0062	0.0992	0.0137	0.0998	0.1215	− 0.0117	0.0480	0.0051	0.0608	0.0660	0.0073	0.0589
250		0.0069	0.0315	0.0612	0.0033	0.0739	0.0093	0.0743	0.0982	− 0.0111	0.0369	0.0033	0.0474	0.0520	0.0047	0.0454
500		0.0048	0.0152	0.0315	0.0027	0.0350	0.0057	0.0352	0.0473	− 0.0053	0.0188	0.0031	0.0219	0.0261	0.0036	0.0215
1000		0.0025	0.0089	0.0141	0.0022	0.0206	0.0037	0.0207	0.0177	− 0.0030	0.0112	0.0019	0.0129	0.0101	0.0023	0.0132
50	$\Xi _{2}$	0.1870	0.2419	0.7330	0.1160	0.4169	0.2461	0.4293	0.4468	− 0.0741	0.2492	0.1144	0.4042	0.4418	0.1515	0.3418
75		0.1118	0.1320	0.3505	0.0810	0.2960	0.1630	0.2964	0.3076	− 0.0795	0.1421	0.0674	0.2159	0.2519	0.0935	0.1924
100		0.0844	0.0991	0.1911	0.0612	0.2229	0.1221	0.2240	0.2392	− 0.0682	0.1073	0.0501	0.1496	0.1528	0.0690	0.1389
125		0.0772	0.0705	0.1486	0.0455	0.1643	0.0941	0.1657	0.2206	− 0.0503	0.0790	0.0403	0.1027	0.1384	0.0573	0.0969
150		0.0499	0.0527	0.1201	0.0244	0.1321	0.0645	0.1332	0.1785	− 0.0594	0.0598	0.0226	0.0812	0.1004	0.0355	0.0783
175		0.0485	0.0512	0.0919	0.0327	0.1243	0.0668	0.1250	0.1335	− 0.0485	0.0573	0.0298	0.0805	0.0695	0.0398	0.0775
200		0.0412	0.0449	0.0706	0.0221	0.0934	0.0519	0.0939	0.1284	− 0.0454	0.0508	0.0219	0.0614	0.0634	0.0303	0.0592
250		0.0424	0.0386	0.0552	0.0337	0.0823	0.0575	0.0827	0.0862	− 0.0296	0.0439	0.0294	0.0533	0.0427	0.0362	0.0529
500		0.0190	0.0192	0.0250	0.0062	0.0360	0.0180	0.0361	0.0428	− 0.0210	0.0227	0.0095	0.0245	0.0207	0.0128	0.0248
1000		0.0064	0.0081	0.0141	0.0011	0.0165	0.0070	0.0165	0.0207	− 0.0159	0.0101	0.0022	0.0107	0.0114	0.0037	0.0110
50	$\Xi _{3}$	0.1055	1.8044	0.0363	− 0.0089	1.3712	0.0494	1.3139	0.0536	− 0.0786	0.9026	0.0374	2.3180	0.0222	0.0554	1.6729
75		0.0725	1.0865	0.0207	0.0254	1.3170	0.0627	1.2600	0.0281	− 0.0589	0.6471	0.0337	1.3431	0.0120	0.0436	1.1255
100		0.0579	0.8065	0.0150	0.0273	1.1212	0.0547	1.0663	0.0225	− 0.0461	0.5064	0.0278	0.9208	0.0099	0.0367	0.8394
125		0.0474	0.5785	0.0130	0.0284	0.9169	0.0505	0.8859	0.0157	− 0.0396	0.3545	0.0270	0.6863	0.0077	0.0322	0.6171
150		0.0349	0.4421	0.0114	0.0169	0.7116	0.0355	0.6918	0.0144	− 0.0395	0.2654	0.0152	0.5039	0.0076	0.0211	0.4690
175		0.0303	0.4028	0.0072	0.0155	0.6241	0.0314	0.6059	0.0093	− 0.0354	0.2454	0.0136	0.4520	0.0037	0.0181	0.4224
200		0.0241	0.3200	0.0077	0.0141	0.5533	0.0280	0.5401	0.0080	− 0.0349	0.1841	0.0120	0.3873	0.0038	0.0152	0.3584
250		0.0220	0.2615	0.0056	0.0154	0.4498	0.0264	0.4363	0.0061	− 0.0275	0.1458	0.0121	0.3016	0.0030	0.0146	0.2846
500		0.0102	0.1329	0.0029	0.0040	0.2026	0.0095	0.1968	0.0034	− 0.0180	0.0642	0.0050	0.1488	0.0016	0.0058	0.1416
1000		0.0058	0.0684	0.0011	0.0030	0.1061	0.0058	0.1033	0.0010	− 0.0104	0.0248	0.0028	0.0736	0.0005	0.0031	0.0703
50	$\Xi _{4}$	0.1069	2.0963	1.1415	− 0.0335	0.8457	0.0670	0.9225	0.4421	− 0.1399	0.6288	0.1102	4.2269	0.8075	0.1248	2.5628
75		0.0652	1.1705	0.5477	− 0.0286	0.6037	0.0332	0.5942	0.3635	− 0.1039	0.4305	0.0631	2.2702	0.4825	0.0828	1.6483
100		0.0513	0.7878	0.2975	0.0025	0.7964	0.0468	0.7672	0.2874	− 0.0806	0.3441	0.0518	1.4137	0.2779	0.0692	1.2163
125		0.0317	0.5673	0.2101	0.0015	0.8547	0.0381	0.8509	0.2617	− 0.0755	0.2705	0.0243	0.9348	0.1979	0.0414	0.8888
150		0.0228	0.4148	0.1734	0.0094	0.8525	0.0404	0.8538	0.2467	− 0.0672	0.2022	0.0169	0.6951	0.1696	0.0309	0.6811
175		0.0296	0.3774	0.1329	0.0217	0.8379	0.0487	0.8441	0.2129	− 0.0493	0.2001	0.0246	0.6179	0.1286	0.0367	0.6147
200		0.0319	0.4053	0.0973	0.0328	0.8788	0.0563	0.8857	0.1630	− 0.0418	0.2217	0.0331	0.6494	0.0848	0.0428	0.6458
250		0.0167	0.2664	0.0743	0.0147	0.6278	0.0335	0.6319	0.1403	− 0.0396	0.1693	0.0135	0.4113	0.0679	0.0216	0.4166
500		0.0113	0.1252	0.0383	0.0104	0.3015	0.0198	0.3038	0.0678	− 0.0179	0.0932	0.0095	0.1904	0.0356	0.0137	0.1987
1000		0.0071	0.0703	0.0161	0.0061	0.1547	0.0108	0.1555	0.0282	− 0.0082	0.0617	0.0060	0.0999	0.0146	0.0082	0.1057
50	$\Xi _{5}$	0.0438	0.1415	2.5457	0.0421	0.4209	0.0766	0.4263	0.6614	− 0.0192	0.1827	0.0312	0.2932	1.2147	0.0392	0.2416
75		0.0261	0.0808	1.5632	0.0261	0.2542	0.0482	0.2571	0.6227	− 0.0188	0.1127	0.0176	0.1585	0.8732	0.0230	0.1407
100		0.0225	0.0605	0.9970	0.0245	0.1922	0.0408	0.1935	0.5640	− 0.0131	0.0866	0.0172	0.1171	0.6180	0.0214	0.1072
125		0.0131	0.0414	0.7249	0.0142	0.1369	0.0271	0.1372	0.4774	− 0.0166	0.0631	0.0092	0.0840	0.4931	0.0129	0.0786
150		0.0123	0.0383	0.4975	0.0099	0.1086	0.0205	0.1085	0.4699	− 0.0130	0.0580	0.0067	0.0650	0.4011	0.0100	0.0639
175		0.0105	0.0295	0.4010	0.0108	0.0901	0.0198	0.0898	0.4384	− 0.0120	0.0470	0.0073	0.0535	0.3502	0.0099	0.0522
200		0.0102	0.0253	0.3340	0.0101	0.0774	0.0180	0.0771	0.3906	− 0.0099	0.0414	0.0073	0.0464	0.2919	0.0095	0.0457
250		0.0087	0.0209	0.2283	0.0085	0.0624	0.0147	0.0620	0.3298	− 0.0079	0.0350	0.0063	0.0371	0.2196	0.0082	0.0372
500		0.0040	0.0113	0.0856	0.0030	0.0298	0.0060	0.0295	0.1576	− 0.0050	0.0200	0.0026	0.0184	0.0826	0.0034	0.0189
1000		0.0021	0.0065	0.0388	0.0022	0.0163	0.0037	0.0161	0.0680	− 0.0028	0.0119	0.0018	0.0104	0.0338	0.0023	0.0109
50	$\Xi _{6}$	0.1666	1.3893	0.0775	− 0.0327	0.8580	0.0539	0.7736	0.1336	− 0.1116	0.5988	0.0815	1.9418	0.0604	0.1135	1.4668
75		0.1149	0.8547	0.0422	0.0245	0.9326	0.0891	0.9454	0.0673	− 0.0852	0.4490	0.0651	1.1122	0.0272	0.0854	0.9698
100		0.0815	0.6172	0.0287	0.0202	0.8205	0.0672	0.8142	0.0436	− 0.0752	0.3468	0.0390	0.7763	0.0182	0.0562	0.7060
125		0.0672	0.4561	0.0230	0.0280	0.7156	0.0646	0.7014	0.0331	− 0.0611	0.2719	0.0340	0.5616	0.0147	0.0463	0.5203
150		0.0535	0.3617	0.0209	0.0213	0.5655	0.0518	0.5541	0.0270	− 0.0566	0.2169	0.0240	0.4155	0.0136	0.0342	0.3938
175		0.0441	0.3020	0.0158	0.0276	0.5523	0.0537	0.5441	0.0195	− 0.0523	0.1890	0.0252	0.3822	0.0091	0.0324	0.3598
200		0.0406	0.2518	0.0152	0.0280	0.4411	0.0511	0.4347	0.0187	− 0.0461	0.1529	0.0235	0.2985	0.0098	0.0306	0.2869
250		0.0239	0.1633	0.0145	0.0150	0.3290	0.0334	0.3236	0.0171	− 0.0471	0.0927	0.0111	0.2079	0.0103	0.0163	0.1993
500		0.0229	0.1144	0.0042	0.0149	0.1782	0.0240	0.1755	0.0066	− 0.0171	0.0769	0.0162	0.1314	0.0026	0.0185	0.1285
1000		0.0102	0.0625	0.0011	0.0072	0.0941	0.0117	0.0929	0.0026	− 0.0129	0.0384	0.0074	0.0702	0.0005	0.0084	0.0694

**Table 2:** The MSEs of all estimators for PNPFD.

*n*	$\Xi$	$\sigma$					$\eta$					$\delta$				
		*MLEs*	*LSEs*	*WLSEs*	*ADEs*	*CVMEs*	*MLEs*	*LSEs*	*WLSEs*	*ADEs*	*CVMEs*	*MLEs*	*LSEs*	*WLSEs*	*ADEs*	*CVMEs*
50	$\Xi _{1}$	0.0350	0.9164	7.2298	0.0602	2.4498	0.0657	2.4846	1.9839	0.0329	0.8452	0.0476	2.0076	3.3589	0.0409	1.3310
75		0.0215	0.2975	2.1777	0.0405	1.0368	0.0430	1.0756	1.3971	0.0211	0.2968	0.0295	0.5478	1.4144	0.0263	0.4365
100		0.0164	0.1959	0.7474	0.0299	0.5978	0.0313	0.6044	0.9718	0.0159	0.2025	0.0214	0.3189	0.7040	0.0197	0.2693
125		0.0125	0.1281	0.3391	0.0222	0.3589	0.0230	0.3623	0.6972	0.0122	0.1315	0.0157	0.1899	0.4454	0.0149	0.1761
150		0.0102	0.0937	0.2158	0.0183	0.2545	0.0189	0.2568	0.5202	0.0101	0.0966	0.0128	0.1343	0.2919	0.0122	0.1259
175		0.0085	0.0754	0.2091	0.0155	0.1979	0.0159	0.1995	0.4544	0.0086	0.0773	0.0107	0.1069	0.2698	0.0102	0.1008
200		0.0074	0.0648	0.1355	0.0140	0.1742	0.0143	0.1753	0.3353	0.0075	0.0666	0.0095	0.0917	0.1792	0.0091	0.0860
250		0.0059	0.0486	0.0944	0.0112	0.1192	0.0113	0.1197	0.2463	0.0060	0.0497	0.0076	0.0687	0.1319	0.0073	0.0650
500		0.0028	0.0203	0.0396	0.0055	0.0481	0.0055	0.0482	0.0874	0.0028	0.0206	0.0035	0.0276	0.0504	0.0035	0.0269
1000		0.0014	0.0101	0.0182	0.0026	0.0210	0.0026	0.0210	0.0381	0.0014	0.0103	0.0017	0.0128	0.0232	0.0017	0.0128
50	$\Xi _{2}$	0.5847	0.9040	7.9696	0.8821	1.3963	0.9744	1.4396	1.8902	0.5390	0.7442	0.7777	2.1126	3.6996	0.6828	1.4891
75		0.3549	0.3591	2.0405	0.6107	0.8756	0.6459	0.8566	1.1688	0.3446	0.3430	0.4777	0.6628	1.4927	0.4310	0.5363
100		0.2503	0.1850	0.8056	0.4479	0.5804	0.4674	0.5763	0.8348	0.2471	0.1856	0.3237	0.3103	0.6678	0.2972	0.2697
125		0.1971	0.1186	0.3572	0.3679	0.3757	0.3806	0.3776	0.6992	0.1929	0.1225	0.2558	0.1887	0.4848	0.2392	0.1707
150		0.1578	0.0933	0.2697	0.3016	0.2867	0.3093	0.2902	0.5161	0.1585	0.0949	0.2060	0.1438	0.3037	0.1958	0.1335
175		0.1407	0.0783	0.1706	0.2559	0.2190	0.2622	0.2204	0.4000	0.1407	0.0801	0.1779	0.1192	0.2208	0.1700	0.1120
200		0.1193	0.0621	0.1284	0.2236	0.1618	0.2282	0.1628	0.3286	0.1195	0.0635	0.1534	0.0906	0.1660	0.1461	0.0838
250		0.0952	0.0480	0.0908	0.1771	0.1162	0.1808	0.1168	0.2212	0.0943	0.0490	0.1198	0.0655	0.1170	0.1161	0.0631
500		0.0449	0.0211	0.0397	0.0826	0.0468	0.0832	0.0469	0.0876	0.0449	0.0214	0.0552	0.0277	0.0507	0.0544	0.0272
1000		0.0227	0.0103	0.0190	0.0410	0.0206	0.0412	0.0206	0.0378	0.0226	0.0102	0.0275	0.0128	0.0235	0.0273	0.0127
50	$\Xi _{3}$	0.2483	45.5009	0.0257	0.2838	17.1766	0.2966	16.6488	0.0453	0.1955	11.9940	0.3091	84.0846	0.0287	0.2641	30.4376
75		0.1552	14.8567	0.0132	0.2274	15.7400	0.2343	15.1950	0.0219	0.1322	7.8209	0.1984	24.5117	0.0150	0.1740	16.1852
100		0.1078	8.2270	0.0091	0.1733	13.2728	0.1768	12.3468	0.0181	0.0944	5.1629	0.1316	11.1046	0.0110	0.1221	9.5963
125		0.0865	5.1812	0.0066	0.1467	10.0372	0.1496	9.7704	0.0107	0.0772	3.6535	0.1062	7.1363	0.0075	0.0992	6.0592
150		0.0686	3.7119	0.0055	0.1160	7.4265	0.1181	7.3836	0.0089	0.0630	2.7863	0.0819	4.7428	0.0062	0.0780	4.3036
175		0.0607	3.1328	0.0043	0.1040	5.8880	0.1055	5.8467	0.0067	0.0555	2.3500	0.0740	3.9161	0.0048	0.0704	3.5930
200		0.0507	2.3945	0.0039	0.0883	4.9424	0.0895	4.9565	0.0058	0.0472	1.8772	0.0621	3.1090	0.0043	0.0590	2.8711
250		0.0416	1.8279	0.0030	0.0735	3.5941	0.0743	3.5261	0.0047	0.0380	1.4362	0.0503	2.2270	0.0034	0.0485	2.1167
500		0.0200	0.7731	0.0015	0.0345	1.3415	0.0346	1.3322	0.0022	0.0182	0.6232	0.0236	0.9131	0.0016	0.0233	0.8944
1000		0.0100	0.3484	0.0007	0.0175	0.5927	0.0175	0.5907	0.0011	0.0087	0.2692	0.0119	0.4079	0.0008	0.0118	0.4031
50	$\Xi _{4}$	0.5394	65.4958	17.9439	0.4029	8.6076	0.4203	9.3527	1.4958	0.3493	7.1522	0.6975	213.1324	10.9343	0.5604	47.7528
75		0.3504	26.0344	5.4808	0.2994	7.0786	0.3060	6.9333	1.0528	0.2428	4.7991	0.4534	90.8851	4.6198	0.3907	26.4526
100		0.2450	13.1107	1.6988	0.2541	8.8115	0.2543	8.4505	0.8205	0.1760	3.7021	0.3076	30.1183	1.7528	0.2804	17.0723
125		0.1920	7.4530	0.9125	0.2449	9.6186	0.2460	9.6347	0.7812	0.1437	2.7665	0.2312	13.0152	0.9789	0.2199	11.0459
150		0.1531	5.1571	0.5582	0.2293	9.6353	0.2313	9.6121	0.7657	0.1161	2.1768	0.1870	8.2455	0.7533	0.1785	7.8311
175		0.1354	4.1452	0.2680	0.2096	9.2349	0.2127	9.2519	0.6569	0.1026	1.8315	0.1633	6.5212	0.4458	0.1576	6.2052
200		0.1229	3.8733	0.1971	0.1966	9.2697	0.1997	9.3258	0.5336	0.0902	1.6708	0.1502	6.2014	0.3029	0.1457	5.9773
250		0.0910	2.4417	0.1343	0.1509	6.1218	0.1526	6.1317	0.4175	0.0703	1.2567	0.1081	3.4860	0.1867	0.1054	3.4159
500		0.0453	1.0639	0.0539	0.0767	2.5467	0.0772	2.5623	0.1406	0.0352	0.6370	0.0539	1.4480	0.0717	0.0532	1.4384
1000		0.0222	0.4686	0.0231	0.0375	1.0304	0.0376	1.0332	0.0560	0.0174	0.3073	0.0261	0.6128	0.0306	0.0260	0.6120
50	$\Xi _{5}$	0.0328	0.3729	44.9797	0.0562	1.4745	0.0624	1.4730	4.4784	0.0312	0.3848	0.0445	0.9783	15.8167	0.0390	0.6693
75		0.0212	0.1405	22.8953	0.0379	0.6566	0.0406	0.6714	4.1388	0.0208	0.1549	0.0280	0.3042	9.4171	0.0256	0.2472
100		0.0156	0.0930	11.3118	0.0281	0.3890	0.0297	0.3980	3.4855	0.0153	0.1017	0.0203	0.1814	5.5097	0.0189	0.1576
125		0.0118	0.0573	7.2812	0.0204	0.2032	0.0212	0.2058	2.9908	0.0119	0.0625	0.0148	0.0997	4.2259	0.0140	0.0904
150		0.0096	0.0459	3.9601	0.0172	0.1614	0.0178	0.1626	2.6388	0.0097	0.0494	0.0121	0.0749	3.0011	0.0117	0.0729
175		0.0082	0.0360	2.4554	0.0147	0.1099	0.0152	0.1107	2.4679	0.0083	0.0385	0.0105	0.0570	2.2836	0.0100	0.0541
200		0.0073	0.0299	1.8674	0.0132	0.0884	0.0135	0.0890	2.0902	0.0073	0.0320	0.0093	0.0468	1.7440	0.0090	0.0446
250		0.0058	0.0224	0.8423	0.0102	0.0630	0.0104	0.0633	1.6791	0.0058	0.0239	0.0071	0.0336	1.1535	0.0070	0.0324
500		0.0027	0.0095	0.2534	0.0050	0.0244	0.0050	0.0245	0.6705	0.0027	0.0099	0.0034	0.0135	0.3736	0.0033	0.0134
1000		0.0014	0.0048	0.1122	0.0025	0.0110	0.0025	0.0110	0.2710	0.0014	0.0048	0.0017	0.0064	0.1502	0.0017	0.0064
50	$\Xi _{6}$	0.5956	26.0656	0.0960	0.6003	9.5584	0.6088	8.7024	0.2159	0.4651	5.8063	0.7404	56.2117	0.1672	0.6500	24.2893
75		0.3782	9.3759	0.0391	0.4895	8.5199	0.5124	8.9862	0.0851	0.3155	3.6691	0.4603	15.1351	0.0474	0.4219	11.1065
100		0.2609	5.3593	0.0261	0.3765	7.2968	0.3887	7.3713	0.0510	0.2257	2.6007	0.3160	8.8636	0.0312	0.2952	6.6803
125		0.2127	3.4845	0.0186	0.3321	6.4983	0.3384	6.4116	0.0356	0.1883	2.0202	0.2548	4.8761	0.0219	0.2416	4.2882
150		0.1736	2.6106	0.0154	0.2708	4.7201	0.2750	4.6522	0.0268	0.1566	1.6672	0.2010	3.1556	0.0176	0.1933	2.9726
175		0.1419	1.9282	0.0124	0.2434	4.3364	0.2474	4.3146	0.0229	0.1301	1.3346	0.1724	2.6186	0.0148	0.1645	2.4318
200		0.1267	1.5839	0.0106	0.2087	3.2011	0.2121	3.1875	0.0182	0.1162	1.1055	0.1479	1.9559	0.0120	0.1430	1.8658
250		0.0963	1.1598	0.0086	0.1664	2.4551	0.1682	2.4409	0.0145	0.0902	0.8845	0.1150	1.4725	0.0098	0.1109	1.3996
500		0.0481	0.4959	0.0038	0.0835	0.9292	0.0841	0.9265	0.0064	0.0444	0.4085	0.0577	0.6031	0.0044	0.0568	0.5881
1000		0.0241	0.2409	0.0019	0.0415	0.4307	0.0417	0.4301	0.0031	0.0220	0.2018	0.0283	0.2843	0.0021	0.0282	0.2818

## Regression analysis

In this section, a novel regression model is presented and serves as an alternative to the Kumaraswamy and beta regression models. The quantile function in Eq. (17) is used to obtain this new regression model. Re-parameterizing the PDF and CDF of the PNPFD can be achieved by utilizing the quantile function. Let $Q\left( p;\sigma ,\eta ,\delta \right) =\mu$ and then

22 $$\begin{aligned} \sigma =\frac{\log \left( \frac{1-\left( 1-p\right) ^{1/\eta }}{1+\delta \left( 1-p\right) ^{1/\eta }}\right) }{\log \left( \mu \right) } \end{aligned}$$

is acquired. The CDF and PDF of the re-parametrized distribution are obtained, respectively, by

23 $$\begin{aligned} F\left( y,\eta ,\delta ,\mu \right) =1-\left( \frac{1-y^{\sigma ^{*}}}{ \delta y^{\sigma ^{*}}+1}\right) ^{\eta }. \end{aligned}$$

and

24 $$\begin{aligned} f\left( y,\eta ,\delta ,\mu \right) =\frac{(\delta +1)\eta \sigma ^{*}y^{\sigma ^{*}-1}\left( \frac{1-y^{\sigma ^{*}}}{\delta y^{\sigma }+1}\right) ^{\eta }}{\left( 1-y^{\sigma ^{*}}\right) \left( \delta y^{\sigma ^{*}}+1\right) }. \end{aligned}$$

where

$$\begin{aligned} \sigma ^{*}=\frac{\log \left( \frac{1-\left( 1-p\right) ^{1/\eta }}{ 1+\delta \left( 1-p\right) ^{1/\eta }}\right) }{\log \left( \mu \right) }, \end{aligned}$$

where parameters $\eta > 0$ and $\delta > -1$ characterize the PNPFD, while $\mu \in (0, 1)$ denotes the quantile regression parameter. The value of *p* is selected from the range (0, 1) and can be either 0.25, 0.5, or 0.75. It is noticed that the random variable *Y* is denoted by $Y \sim PNPF\left( \eta ,\delta ,\mu ,p\right)$.

Once the QPNPF has been defined, the new regression model using the PDF of the QPNPF in Eq. (24) can be presented. Let $y_{1},y_{2},\ldots ,y_{n}$ such that $y_{i}$ is an realization of $Y^{\tilde{\phantom{a}}}QPNPF\left( \eta ,\delta ,\mu _{i},p\right)$ for $i=1,2,\ldots ,n$ where $\eta ,\delta$ and $\mu _{i}$ are unknown parameters, and the *p* is known. The proposed quantile regression model is as follows:

25 $$\begin{aligned} g\left( \mu _{i}\right) ={\textbf{x}}_{i}\mathbf {\beta }^{\texttt{T}}, \end{aligned}$$

where $\mathbf {\beta }\mathbf {=}\left( \beta _{0},\beta _{1},\ldots ,\beta _{p}\right)$ are the unknown regression parameter vector, ${\textbf{x}} _{i}=\left( \textbf{1,x}_{i1},{\textbf{x}}_{i2},\ldots ,{\textbf{x}}_{ip}\right)$ known *i*th vector of the covariates and *g* is a link function. We use the following logit-link function because the QPNPF is defined within the interval (0, 1):

26 $$\begin{aligned} g\left( \mu _{i}\right) =\log \left( \frac{\mu _{i}}{1-\mu _{i}}\right) ,i=1,2,\ldots ,n. \end{aligned}$$

It is achieved by Eq. (26)

27 $$\begin{aligned} \mu _{i}=\frac{\exp \left( {\textbf{x}}_{i}\mathbf {\beta }^{\texttt{T}}\right) }{1+\exp \left( {\textbf{x}}_{i}\mathbf {\beta }^{\texttt{T}}\right) }. \end{aligned}$$

### Parameter estimation for regression parameters

In this section, for the estimate of unknown regression parameters and model parameters, the maximum likelihood estimation method is introduced. Let $Y_{1},Y_{2},\ldots ,Y_{n}$ be a random sample of size *n* from the $QPNPF\left( \eta ,\delta ,\mu _{i},p\right)$ distribution with realizations $y_{1},y_{2},\ldots ,y_{n}$, where the $\mu _{i}$ is given in (27) for $i=1,2,\ldots ,n.$Then the log-likelihood function is given by

28 $$\begin{aligned} \ell \left( \Xi \right)= & {} n\log \left( \delta +1\right) +n\log \left( \eta \right) +n\log \left( \sigma ^{*}\right) +\left( \sigma ^{*}-1\right) \sum \limits _{i=1}^{n}\log \left( y_{i}\right) \nonumber \\{} & {} +\eta \sum \limits _{i=1}^{n}\log \left( \frac{1-y^{\sigma ^{*}}}{\delta y^{\sigma ^{*}}+1}\right) -\sum \limits _{i=1}^{n}\log \left( 1-y_{i}^{\sigma ^{*}}\right) -\sum \limits _{i=1}^{n}\log \left( \delta y_{i}^{\sigma ^{*}}+1\right) \end{aligned}$$

where $\Xi =\left( \eta ,\delta ,\mathbf {\beta }\right)$ is the parameter vector. The MLE of the $\Xi ,$ say ${\widehat{\Xi }}=\left( {\widehat{\eta }}, {\widehat{\delta }},\beta _{0},\beta _{1},\ldots ,\beta _{p}\right)$ is achieved by maximizing the $\ell \left( \Xi \right)$ presented in (28) for $\eta ,\delta$ and $\mathbf {\beta .}$ As the log-likelihood function in (28) involves a nonlinear function, and it can be maximized using **optim** function in R.

## Real data analysis

In this section, three real data applications are examined for both the proposed distribution and novel regression model.

### Practical examples for PNPFD

In this subsection, two practical data sets are analyzed to demonstrate the usability of the PNPFD. The Kumaraswamy (K)^24^, unit-Weibull (UW)^25^, unit-Burr XII (UBXII)^26^, unit-Muth(UM)^27^, and NPFD models are used to compare the PNPFD. The PDFs for these models are given, respectively, by

$$\begin{aligned} f_{PNPFD}\left( y\right)= & {} \frac{\left( p_{2}+1\right) p_{3}p_{1}y^{p_{1}-1}\left( \frac{1-y^{p_{1}}}{1+y^{p_{1}p_{2}}}\right) ^{p_{3}}}{(1-y^{p_{1}})(p_{2}y^{p_{1}}+1)},p_{1},p_{3}>0,p_{2}>-1.\\ f_{K}\left( y\right)= & {} p_{1}p_{2}y^{p_{1}-1}\left( 1-y^{p_{1}}\right) ^{p_{2}-1},p_{1},p_{2}>0.\\ f_{UW}\left( y\right)= & {} p_{1}p_{2}\left( -log\left( y\right) \right) ^{p_{2}-1}\exp \left( -p_{1}\left( -log\left( y\right) \right) ^{p_{2}}\right) y^{-1},p_{1},p_{2}>0.\\ f_{UBXII}\left( y\right)= & {} p_{1}p_{2}y^{-1}\left( -\log y\right) ^{p_{2}-1}\left( 1+\left( -\log y\right) ^{p_{2}}\right) ^{-p_{1}-1},p_{1},p_{2}>0.\\ f_{UM}\left( y\right)= & {} p_{2}^{-1}\exp \left( 1/p_{1}\right) \left( y^{-\frac{ p_{1}}{p_{2}}}-p_{1}\right) y^{-1-\frac{p_{1}}{p_{2}}}\exp \left( -\frac{1}{ p_{1}}y^{-\frac{p_{1}}{p_{2}}}\right) ,p_{1},p_{2}>0.\\ f_{NPFD}\left( y\right)= & {} \left( p_{1}+1\right) p_{2}\left( 1-y\right) ^{p_{2}-1}\left( 1+p_{1}y\right) ^{-p_{2}-1},p_{1},p_{2}>0. \end{aligned}$$

The maximum likelihood methodology is used to estimate the model parameters. The estimated log-likelihood ($\ell$), Akaike information criterion (AIC), and the Bayesian information criterion (BIC) are used to assess the goodness-of-fit of the distributions. Furthermore, the Kolmogrov-Smirnov (KS) statistic and p-value of the KS statistic are calculated.

The first set of data was taken from firm risk management cost-effectiveness, which is available on the web page of Professor E. Frees (Wisconsin School of Business). The data is defined on (0, 1) and calculated as the total property and casualty premiums and uninsured losses as a percentage of the total assets. The first data is also reported and analyzed by^34^. Table 3 reports the first real data set modeling results.

**Table 3:** The goodness of fit results for the first data sets.

	$p_{1}$	$p_{2}$	$p_{3}$	$\ell$	AIC	BIC	CAIC	HQIC	KS	*p*-value
PNPFD	1.4640	63.2352	0.9336	93.6619	− 181.3238	− 1173.5083	− 1181.0738	− 178.1607	0.0629	0.9350
K	0.6648	3.4407	–	78.6539	− 1153.3079	− 1148.0975	− 1153.1841	− 1151.1991	0.1535	0.0642
UW	0.0653	2.3529	–	88.1005	− 1172.2010	− 1166.9907	− 1172.077	− 1170.0923	0.0931	0.5516
UBXII	0.3482	2.8408	–	46.5066	− 189.0132	− 183.8029	− 188.8895	− 86.9045	4.5609	0.0000
UM	0.9390	2.8142	–	89.9609	− 1175.9217	− 1170.7114	− 1175.7980	− 173.8130	0.1086	0.3550
NPFD	8.9785	1.3829	–	90.5154	− 1177.0307	− 1171.8204	− 1176.9070	− 174.9220	0.1029	0.4223

The second data set indicates the recovery rates of viable CD34+ cells in the 239 patients who agreed to autologous peripheral blood stem cell transplant after myeloablative chemotherapy doses. The CD34+ is also investigated by^26^. Results for the CD34+ are given in Table 4.

**Table 4:** The goodness of fit results for the second data sets.

	$p_{1}$	$p_{2}$	$p_{3}$	$\ell$	AIC	BIC	CAIC	HQIC	KS	p-value
PNPFD	8.5683	2.3550	1.4693	194.6930	− 1383.3860	− 1375.5704	− 1383.1360	− 1380.2229	0.0404	0.8311
K	6.6942	2.4355	–	190.7640	− 1377.5280	− 1372.3176	− 1377.4043	− 1375.4193	0.0723	0.1646
UW	8.0563	1.6182	–	192.0157	− 1380.0314	− 1374.8211	− 1379.9077	− 1377.9227	0.0557	0.4487
UBXII	10.0756	1.7320	–	193.5027	− 1383.0054	− 1377.7951	− 1382.8817	− 1380.8967	1.9997	0.0000
UM	0.4900	0.2478	–	179.9752	− 1355.9504	− 1350.7401	− 1355.8267	− 1353.8417	0.0869	0.0539
NPFD	-0.9570	4.2844	–	139.5322	− 1275.0644	− 1269.8541	− 1274.9407	− 1272.9557	0.1891	0.0000

**Figure 3:**
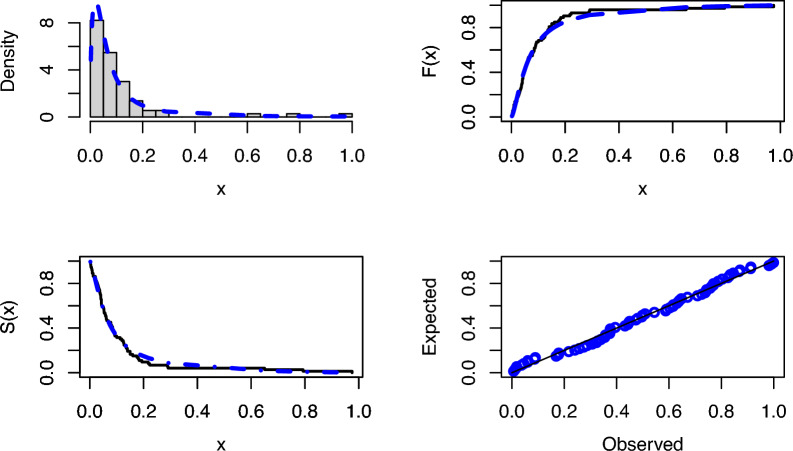
The fitted PDF, CDF, SF, and P-P plots for PNPFD of the first data.

**Figure 4:**
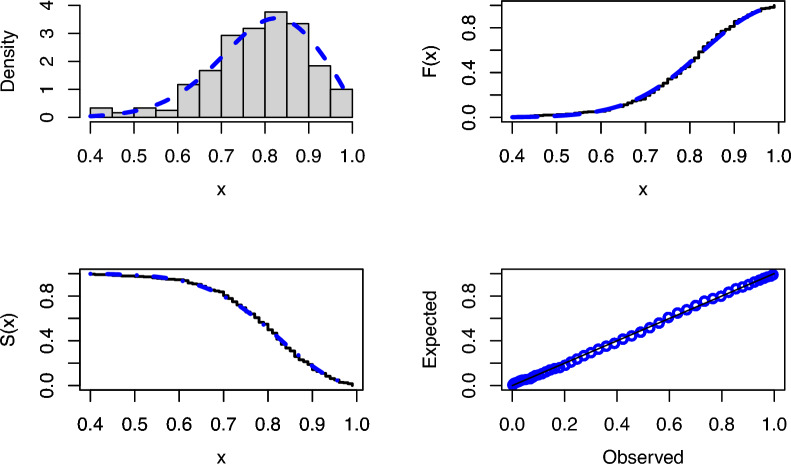
The fitted PDF, CDF, SF, and P-P plots for PNPFD of the second data.

When the modeling results for both real data sets are analyzed, Tables 3 and 4 clearly show that PNPFD is the best model among all models based on all criteria and statistics. Figures 3 and 4 present some goodness-of-fit graphs for real data modeling. In Figures 3 and 4, the fitted PDF, CDF, SF, and P-P plots of the PNPFD based on the first and second real datasets are illustrated in detail. Considering the fit in Figures 3 and 4, it is observed that the PNPFD is a suitable choice for modeling these two real datasets.

### Practical example for QPNPFD

In this subsection, the new regression model is demonstrated for its usability through a real data application. For comparison purposes, the Kumaraswamy^30^ and the beta^29^, log-extended exponential geometric (LEEG)^35^, and transmuted unit rayleigh (TUR)^36^ regression models are utilized. The quantile parameter *p* is set to 0.5 for the QPNPFD, Kw, and LEEG regression models. The data is taken from^36^ and can be found at https://stats.oecd.org/index.aspx?DataSetCode=BLI. Here, the percentage of the educational attainment values of the OECD countries (*y*) is considered as the dependent variable, and the percentage of the voter turnout ($x_{1}$), homicide rate ($x_{2}$), and life satisfaction ($x_{3}$) as the independent variables. Detailed information about this data and some descriptive statistics can be viewed from^36^. This application aims to reveal the relationship with *y* and $x_{1}$, $x_{2}$, and $x_{3}$.

The regression model is presented as

$$\begin{aligned} \text {logit} \left( \mu _{i}\right) =\beta _{0}+\beta _{1}x_{i1}+\beta _{2}x_{i2}+\beta _{3}x_{i3},\text { }i=1,2,\ldots ,38. \end{aligned}$$

where $\mu _{i}$ represents the median for QPNPFD, Kw, and LEEG models and the mean for Beta regression. Parameter estimates for regression models, p-values for the significance of model parameters, and log-likelihood results are presented in Table 5.

**Table 5:** Parameter estimates of regression models for OECD data with standard error (SE) and log-likelihoods.

Parameters	PNPFD			TUR			Beta			Kumaraswamy			LEEG		
	MLE	SE	p-value	MLE	SE	p-value	MLE	SE	p-value	MLE	SE	p-value	MLE	SE	p-value
$\beta _{0}$	0.7746	1.2807	0.5453	− 0.3469	1.0407	0.7389	0.9615	0.9685	0.3208	1.6247	1.1740	0.1664	0.3275	1.0754	0.7607
$\beta _{1}$	$-$2.4192	1.0693	0.0237	− 1.7892	0.8534	0.0360	− 2.9211	1.0176	0.0041	− 4.1197	1.3892	0.0030	− 4.0917	1.4520	0.0048
$\beta _{2}$	$-$0.0604	0.0273	0.0269	− 0.0673	0.0237	0.0046	− 0.0470	0.0178	0.0084	− 0.0404	0.0168	0.0159	− 0.0477	0.0145	0.0010
$\beta _{3}$	0.3814	0.1637	0.0198	0.4754	0.1356	–	0.3794	0.1492	0.0110	0.4237	0.2546	0.0960	0.6214	0.1745	$< 0.001$
$\eta$	3.9360	4.5452	0.3865	−	−	−	−	−	−	−	−	−	−	−	−
$\delta$	−0.2720	1.3055	0.8350	−	−	−	−	−	−	−	−	−	−	−	−
$\alpha$	−	−	−	-0.4272	0.5474	0.4352	11.5900	2.6100	–	6.2167	1.0787	–	7.8378	1.7365	–
${\widehat{\ell }}$	33.2672			32.9941			30.9024			29.4339			28.6480		

From 5, it is striking that the best regression model for OECD data is the PNPFD model. For the PNPFD model, $\eta$, $\delta$, and $\beta _{0}$ parameters are statistically insignificant at the level of 5%, and the other parameters $\beta _{1}$, $\beta _{2}$ and $\beta _{3}$ are statistically significant at the level of 5%. The median response is positively affected by parameter $\beta _{3}$, whereas the median response is negatively affected by parameters $\beta _{1}$ and $\beta _{2}$. It is determined that an increase in life satisfaction increases the percentage of educational attainment, while an increase in voter turnout and homicide rate decreases the percentage of educational attainment.

## Conclusion

This study aimed to introduce a new superior model capable of modeling and fitting data defined on (0,1). This paper introduced a new unit model as an alternative to Kumaraswamy and beta distributions. The new model’s statistical and reliability features were discussed, like moments, stochastic ordering, reliability function, hazard rate function, order statistics, and quantile function. Furthermore, the PNPFD has flexible shapes for its density and hazard functions. The probability density function plots reveal that the new distribution is unimodal and J-shaped, while the hazard rate function exhibits a pattern characterized by decreased, increased, and bathtub-shaped behavior. The major objectives had been established throughout the study, setting the groundwork for a comprehensive investigation into the efficacy of the PNPFD compared to existing, well-known distributions. As we delve into the conclusion, it is noteworthy to emphasize that the research aim has been realized with resounding success. Its parameters are estimated with precision using various methods. The performance of these methods is compared with a Monte Carlo simulation. According to the simulation study, it is observed that the results of the estimators approached each other in a large sample size. Simulation results indicate that, according to the bias criterion, ADEs are typically identified as the optimal estimator for the parameters of $\sigma$ and $\eta$, while MLEs are considered the most suitable estimator for the $\delta$ parameter.. A novel regression analysis is introduced via the proposed distribution. Three real data analyses demonstrate the applicability and reliability of the new distribution and the new regression model evidenced by low error measures such as SE and p-value. The results from the modeling with figures also demonstrate that the new distribution fits remarkably well with the real data. In conclusion, this study not only ensued in meeting its aim but also proved the capability of the PNPFD to contribute substantially to the field of statistics. The flexibility of the proposed regression model compared to existing regression models indicates that it is an effective model for situations where the dependent variable is proportional. The outcomes portrayed here open paths for future research incorporating novel heuristics techniques for investigating the disease dynamics and insist on the significance of the PNPFD as a beneficial tool for researchers in diverse areas, including neuro-computational intelligence, non-linear tumor-immune delayed model, nonlinear multi-delayed tumor oncolytic virotherapy systems, nonlinear influenza-A epidemic model, nonlinear multi-delays SVEIR epidemic systems, etc. We hope that this model will be used for data analysis in many different fields such as economics, engineering, medicine, etc. In addition to the properties we have discussed, several other methods, such as Bayesian regression and the method of moments, can be employed to estimate parameters to assess the efficiency of a model. By applying these methods, we can make future predictions based on the data set, allowing for further analysis and application of the proposed model.

## Data Availability

All data exists in the paper with its related references.
